# Trajectories of Body Mass Index and Multimorbidity in Old Age: 12-Year Results From a Population-Based Study

**DOI:** 10.1093/geroni/igab046.201

**Published:** 2021-12-17

**Authors:** Amaia Calderón-Larrañaga, Xiaonan Hu, Jie Guo, Luigi Ferrucci, Weili Xu, Davide Vetrano

**Affiliations:** 1 Karolinska Institutet, Solna, Stockholms Lan, Sweden; 2 National Institute on Aging, Baltimore, Maryland, United States

## Abstract

We aimed to study the association of long-terms trajectories of body mass index (BMI) with contemporaneous changes in multimorbidity development in older adults. Twelve-year BMI trajectories (2001–2013) were identified in subjects aged 60+ years from the Swedish National Study on Aging and Care-Kungsholmen (SNAC-K) using growth mixture models (N=2,189). Information on chronic diseases and multimorbidity was ascertained based on clinical examinations, lab tests, medications, and inpatient and outpatient medical records. Linear mixed models were used to study the association between BMI trajectories and the speed of chronic diseases accumulation, in general and by groups of cardiovascular and neuropsychiatric diseases. Eighty percent of the study population was included in a stable BMI trajectory, 18% in a slow-decline trajectory with an accelerated BMI decline from age 78 onwards, and 2% in a fast-decline trajectory that reached underweight values before age 85. A significantly higher yearly rate of chronic disease accumulation was observed in the fast-decline versus stable trajectories (β=0.221, 95% CI 0.090-0.352) after adjusting for age, sex, education and time to death. Subjects in the slow-decline trajectory showed a significantly higher rate of cardiovascular diseases accumulation (β=0.016, 95% CI 0.000-0.031); those in the fast-decline trajectory showed a faster accumulation of both cardiovascular (β=0.020, 95% CI -0.025, 0.064) and neuropsychiatric diseases (β=0.102, 95% CI 0.064-0.139), even if the former association did not reach statistical significance. Carefully monitoring older adults with sustained weight loss seems relevant given their likelihood to develop a phenotype of rapidly accumulating chronic -especially neuropsychiatric- diseases.

